# A Methyl-Modified Silica Layer Supported on Porous Ceramic Membranes for the Enhanced Separation of Methyl Tert-Butyl Ether from Aqueous Solution

**DOI:** 10.3390/membranes12050452

**Published:** 2022-04-22

**Authors:** Ligang Xu, Yali Wang, Qunyan Li, Suping Cui, Mingxue Tang, Zuoren Nie, Qi Wei

**Affiliations:** 1 Faculty of Materials and Manufacturing, Beijing University of Technology, 100 Pingleyuan, Chaoyang District, Beijing 100124, China; xulg@emails.bjut.edu.cn (L.X.); qyli@bjut.edu.cn (Q.L.); cuisuping@bjut.edu.cn (S.C.); zrnie@bjut.edu.cn (Z.N.); 2 Center for High Pressure Science and Technology Advanced Research, Beijing 100094, China; mingxue.tang@hpstar.ac.cn

**Keywords:** methyl-modified silica layer, one-pot synthesis, hydrophobicity, pervaporation, MTBE/water separation performance, long-term stability

## Abstract

As a kind of volatile organic compound (VOC), methyl tert-butyl ether (MTBE) is hazardous to human health and destructive to the environment if not handled properly. MTBE should be removed before the release of wastewater. The present work supported the methyl-modified silica layer (MSL) on porous α-Al_2_O_3_ ceramic membranes with methyltrimethoxysilane (MTMS) as a precursor and pre-synthesized mesoporous silica microspheres as dopants by the sol-gel reaction and dip-coating method. MTMS is an environmentally friendly agent compared to fluorinated alkylsilane. The MSL-supported Al_2_O_3_ ceramic membranes were used for MTBE/water separation by pervaporation. The NMR spectra revealed that MTMS evolves gradually from an oligomer to a highly cross-linked methyl-modified silica species. Methyl-modified silica species and pre-synthesized mesoporous silica microspheres combine into hydrophobic mesoporous MSL. MSL makes the α-Al_2_O_3_ ceramic membranes transfer from amphiphilic to hydrophobic and oleophilic. The MSL-supported α-Al_2_O_3_ ceramic membranes (MSL-10) exhibit an MTBE/water separation factor of 27.1 and a total flux of 0.448 kg m^−2^ h^−1^, which are considerably higher than those of previously reported membranes that are modified by other alkylsilanes via the post-grafting method. The mesopores within the MSL provide a pathway for the transport of MTBE molecules across the membranes. The presence of methyl groups on the external and inner surface is responsible for the favorable separation performance and the outstanding long-term stability of the MSL-supported porous α-Al_2_O_3_ ceramic membranes.

## 1. Introduction

Volatile organic compounds (VOCs) are widely used in the printing industry, refrigeration, and petrochemical production [1,2,3]. The release of wastewater that VOCs contaminate causes serious harm to human health and the ecological environment [4]. Methyl tert-butyl ether (MTBE), one of the hazardous VOCs, shows a maximum solubility of 5.1 wt% in water and is frequently detected in wastewater [5]. It is of great significance to separate VOCs, including but not limited to MTBE, from wastewater [3,6,7]. Among all separation techniques, membrane separation based on pervaporation (PV) has drawn significant attention due to relatively mild operating conditions such as room temperatures, low pressures, medium cross-flow velocities, and unnecessary additional chemicals [8,9,10,11,12,13]. It is generally believed that some polymers are chemically stable, intrinsically hydrophobic, and relatively low-cost [14,15]. Hydrophobic polymer pervaporation membranes are also suitable for solving the problem of separating MTBE from wastewater [16]. However, ceramic membranes have their own distinct merits compared to polymer membranes, such as uncomplicated cleaning, excellent non-swelling behavior, high mechanical strength, and anti-fouling property [6,17,18,19,20,21,22,23,24,25,26]. Ceramic membranes must be hydrophobized before the PV separation due to their inherently hydrophilic nature [27,28,29,30,31]. The post-grafting method has been widely used for hydrophobization by replacing hydrophilic hydroxyl groups on the membrane with hydrophobic groups [9,30,32,33].

In the last decades, fluoroalkylsilane (FAS) molecules were intensively adopted as modifiers for PV due to their high selectivity toward VOCs [10,20,30,32,33]. Although FAS molecules exhibit great affinity to VOCs, nonfluorinated alkylsilane (NFAS) molecules have more potential in either the synthesis process or practical applications if considering the environmental issue, because excessive fluoride in the environment has proved to cause various human diseases [34]. In our previous work, silica membranes were modified by different NFAS (octyltriethoxysilane (OTES) or propyltriethoxysilane (PTES)) via a post-grafting strategy. The OTES- and PTES-modified silica membranes show an MTBE/water separation factor of 15.3 and 12.0, respectively, considerably lower than that of the membranes modified by perfluorooctyltriethoxysilane (PFOTES) (24.6) [35]. This observation might be related to the fact that the NFAS molecules are less compatible with MTBE than the FAS molecules due to the relatively larger surface free energy of NFAS [36]. Hence, the MTBE molecules dissolve with more difficultly in the NFAS-modified membranes, thus resulting in an inferior MTBE/water separation performance.

Increasing the loading of nonfluorinated alkyl chains on the membrane surface is probably a promising strategy to enhance the VOCs separation performance. However, it has been proved that the post-grafting approach leads to a relatively low loading of the functional groups [37]. Direct use of NFAS such as methyltrimethoxysilane (MTMS) as a membrane precursor, known as a one-pot synthesis method, may result in a higher loading of functional groups in the final membranes [37].

This work proposed a novel one-pot synthesis method to prepare methyl-modified silica layer (MSL) supported porous α-Al_2_O_3_ ceramic membranes. Methyl-modified silica sol was prepared via a sol-gel reaction using MTMS, which has a short carbon chain (*CH*_3_) in the molecule, as both precursor and modifier. Pre-prepared mesoporous silica microspheres were doped into the sol, and then the composite sol was dip-coated on α-Al_2_O_3_ ceramic membranes. The structural evolution of MTMS in the critical steps of the sol-gel reaction was tracked by locally sensitive nuclear magnetic resonance (NMR) characterization. The MSL-supported porous Al_2_O_3_ ceramic membranes were used to separate MTBE from aqueous solution, and the separation mechanism and the long-term stability were also discussed in depth. The one-pot strategy for designing modified membranes with improved separation performance would extend to broader related applications.

## 2. Materials and Methods

### 2.1. Materials

Aluminum oxide (Al_2_O_3_, 99.99%), starch soluble (98%), methyltrimethoxysilane (MTMS, 98%), hydrochloric acid (HCl, 37%), absolute ethanol (EtOH, 99.9%), polymethyl methacrylate (PMMA, 97%) and methyl tert-butyl ether (MTBE, 99%) were obtained from Fuchen Chemical Reagent Factory (Tianjin, China). Deionized water (H_2_O) with a resistivity close to 10Ω cm was produced by a water purification system (Ulupure, Chengdu, China). Silica microspheres were home-made according to the references [38,39].

### 2.2. Fabrication of MSL-Supported α-Al_2_O_3_ Ceramic Membranes

**Figure 1:**
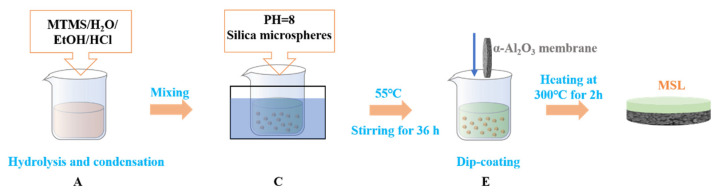
Fabrication process of the MSL-supported α-Al_2_O_3_ ceramic membranes.

The preparation of MSL-supported α-Al_2_O_3_ ceramic membranes is illustrated in Figure 1. Methyltrimethoxysilane, ethanol, and deionized water were mixed in a molar ratio of 3:7:2 at room temperature and then stirred at 55 °C for an additional 2 h to obtain mixture A. Then, 42 mL of mixture A was cooled to room temperature, followed by the addition of 4 mL of 0.1 mol L^−1^ hydrochloric acid and 4 mL of deionized water successively. After stirring at 30 °C for 0.5 h, the solution was aged at 55 °C for 24 h to achieve mixture B. Afterward, mixture B was adjusted to a pH of 8.0 to accelerate the condensation reaction [40] and denoted as mixture C. The silica microspheres were dispersed in ethanol with a weight percentage of 3% to yield mixture D. The mixture D was then mixed with C in a volume ratio of 1:1 and stirred at 55 °C for 36 h to generate mixture E. The MSL was supported on porous α-Al_2_O_3_ ceramic membranes (see the Supplementary Material S1, Figure S1 for the preparation and pore structure) by dip-coating using a dip-coater (KSV, Finland) as follows. The α-Al_2_O_3_ ceramic membrane was dipped into mixture E for 3 s with an immersing and withdrawing rate of 80 mm min^−1^, and then dried at room temperature for 5 min. The dip-coating and drying procedure was repeated 5, 10 and 15 times, respectively. The coated samples were heated at 300 °C for 2 h with a ramping rate of 0.5 °C min^−1^, and the final products were denoted as MSL-5, MSL-10, and MSL-15, respectively, depending on the dip-coating cycles. The residual mixture E was dried and then heated in the same manner as the coated samples for N_2_ adsorption measurements.

### 2.3. Material Characterization

The surface and cross-sectional morphology of the samples was observed by scanning electron microscopy (SEM) (Gemini SEM 300). Gold was sputtered on the samples to ensure conductivity. The contact angle of different liquids was detected on the video-based contact angle measurement system (Dataphysics OCA20). A droplet of 3 μL was injected on the surface of the membrane at a rate of 1 μL/s. The surface roughness was measured by atomic force microscopy (AFM) (Bruker Dimension Icon). The pore size distribution of the α-Al_2_O_3_ ceramic membrane was determined by the bubble point method on the 3H–2000 PB capillary flow porometer (BeiShiDe Instrument, Beijing, China). The pore structure of silica microspheres and unsupported MSL was determined on a volume N_2_ adsorption analyzer (Micromeritics ASAP 2020M). The Brunauer–Emmett–Teller (BET) method was used to evaluate the surface area. The pore size distribution was calculated from the desorption branch of the isotherm using the Barrett–Joyner–Halenda (BJH) approach. The nitrogen permeation and liquid entry pressure (LEP) were measured using a home-made setup. For the nitrogen permeation, the transmembrane pressure was fixed at 100 kPa by a gas regulator valve, and the permeate nitrogen fluxes were measured by a gas mass flowmeter (Alicat Scientific, Tucson, AZ, USA). For the LEP measurement, water was fed into the membrane chamber by pressurized nitrogen, and the weight of the permeate water was measured by an electronic balance (Mettler-Toledo, Shanghai, China). Nuclear magnetic resonance (NMR) measurements were performed on a Bruker 400 MHz spectrometer (AVANCE HD III). The Larmor frequencies for ^13^C and ^29^Si were 100.6 and 79.5 MHz, respectively. All the samples were filled into 4.0 mm rotors. The magic angle spinning (MAS) rate was set to 8 kHz. All spectra were obtained by using a single pulse to polarize. The pulse lengths of 2.5 and 2.9 µs were used to excite ^13^C and ^29^Si signals, respectively. High power decoupling was applied to remove the coupling influence of protons when acquiring a ^13^C signal [41].

### 2.4. Vacuum Pervaporation

As shown in Figure S2, the pervaporation experiment was carried out at 30 °C on a home-made vacuum pervaporation device. The membrane was sealed in a stainless steel module by silastic rings. The MTBE aqueous solution was continuously cycled to the feed side of the membrane by a peristaltic pump. A vacuum pump was used to produce transmembrane pressure at the permeate side. The vapor generated at the permeate side was collected by a cold trap cooled with liquid nitrogen. The collected permeate was weighed by an electronic balance, and the concentration of MTBE in the permeate was analyzed by a gas chromatograph (GC-2014 AT, Shimadzu) equipped with a thermal conductivity detector and a Porapak Q column, using helium as carrier gas. The MTBE/water permeate was dissolved in acetone to obtain a homogenous solution for composition analysis. The permeate flux (J) and the MTBE/water separation factor (α) were evaluated by Equations (1) and (2), respectively [11]. Multiplying the total fluxes (J) by the concentration of the components, which the GC measurement can determine, will obtain the partial fluxes of the components.

(1) $$J=\frac{M}{A\times t}\left[\mathrm{kg}\;m^{-2}\;h^{-1}\right]\;$$

(2) $$\alpha =\frac{y_{0}/y_{w}}{x_{0}/x_{w}}\;$$

where *M* represents the total permeate weight (kg), *A* is the effective membrane area (m^2^), *t* refers to the pervaporation time (h), $y_{0}$ and $\;y_{w}$ mean the mass percentage of MTBE and water in the permeate, and $x_{0}$ and $x_{w}$ denote the mass fraction of MTBE and water in the feed, respectively.

## 3. Results and Discussion

### 3.1. Evolution of MTMS Precursor and Formation of MSL during the Sol-Gel Reaction

**Figure 2:**
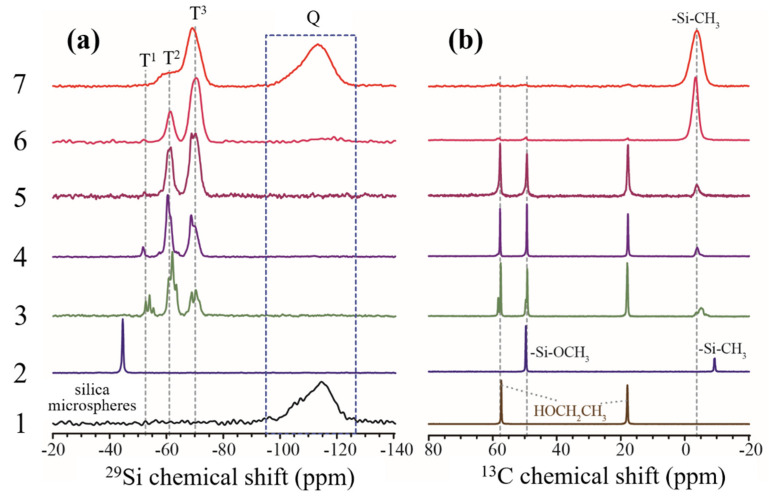
High-resolution ^29^Si NMR (**a**) and ^13^C NMR (**b**) spectra for tracking the evolution of MTMS precursors and the formation of MSL. The label 1–7 denotes the precursor, solvent, or the products generated at each step of the sol-gel reaction: 1. silica microsphere (**a**) or ethanol (**b**); 2. methyltrimethoxysilane; 3. mixture A in Section 2.2; 4. mixture B in Section 2.2; 5. mixture C in Section 2.2; 6. mixture E in Section 2.2, after drying; 7. sample 6 after sintering at 300 °C for 2 h.

NMR is sensitive to probing the local structure information and is employed to detect the chemical environments of the intermediate products during the sol-gel reaction. For reference, the solid-state ^29^Si NMR spectrum of silica microspheres is also measured and plotted in Figure 2(a1). A broad peak corresponding to the resonances of Q [Si(OSi)_4-n_(OH)_n_, n ≤ 3] silicon atoms is observed at the chemical shift ranging from −95 to −115 ppm [39]. As shown in Figure 2(a2), the ^29^Si NMR spectrum of MTMS precursor exhibits a sharp peak at −44.7 ppm, indicating that there is only one ^29^Si environment [CH_3_Si(OCH_3_)_3_] for MTMS as expected (See Figure S3a for the illustration). As depicted in Figure 2(a3), after reaction at a temperature of 55 °C for 2 h, the peak of the pristine MTMS disappears, while new peaks, assigned to *T*^1^ [CH_3_Si(OSi)(OCH_3_)_2_, CH_3_Si(OSi)(OCH_3_)OH or CH_3_Si(OSi)(OH)_2_], T^2^ [CH_3_Si(OSi)_2_(OH)] and *T*^3^ (CH_3_Si(OSi)_3_) silicon atoms, appear at the chemical shift of −54.1, −62.1 and −70.4 ppm, respectively (See Figure S5b for the illustration) [42,43]. The absence of the silicon signal of MTMS and the presence of *T*^1^, *T*^2^ and *T*^3^ resonances indicate that hydrolysis and condensation occur simultaneously, resulting in various oligomers. Obviously, the condensation degree of oligomers increases in the order of *T*^1^ < *T*^2^ < *T*^3^, as demonstrated by the fact that the number of bridged oxygen per oligomer increases gradually. After aging for 24 h, the *T*^1^ and *T*^2^ peak intensity decrease, but that of the *T*^3^ peak increases, suggesting that the condensation rate is higher than the hydrolysis rate during the aging process (Figure 2(a4)). While adjusting the pH to 8, the *T*^1^ and *T*^2^ peaks continue to become weaker, but the *T*^3^ peak turns stronger, revealing that the alkaline environment favors condensation (Figure 2(a5)) [44]. The drying leads to further condensation, as demonstrated by the reduced intensity in the *T*^1^ and *T*^2^ peaks (Figure 2(a6)). After calcination at 300 °C, the *T*^3^ peak becomes absolutely predominant. At the same time, the *T*^1^ signal disappears entirely, and the *T*^2^ peak becomes much weaker (Figure 2(a7)), indicating that a highly cross-linked network is formed (see Figure S3c for the illustration). Compared to Figure 2(a6), the *Q* silicon signal derived from the doping silica microspheres can be detected in Figure 2(a7) since the measurement is carried out under magic angle spinning (MAS), which is more applicable for solid samples. The spectral resolution for a solid sample is usually less than that of a liquid sample, although MAS is used in the NMR measurement. Hence, a broader peak in Figure 2(a7) is observed compared to other samples.

To consolidate the above process, ^13^C NMR spectra are performed and analyzed as well (Figure 2b). Two resonances at 57 ppm and 18 ppm are observed for the -O^13^CH_2_ and -^13^CH_3_ groups of ethanol solvent, respectively. The solvent signal presents in the following reaction steps and disappears during the drying and sintering processes (Figure 2(b6,b7)). The ^13^C peak in -Si-^13^CH_3_ appears upfield, possibly due to the annular structure in the chemical environment [45]. As shown in Figure 2(b2), the pristine MTMS shows two carbon signals at 49.6 and −9.4 ppm for -Si-O^13^CH_3_ and -Si-^13^CH_3_, respectively. In the following sol-gel processes, the peak at 49.6 ppm remains unchanged, indicating that -OCH_3_ groups are not entirely consumed by the hydrolysis and condensation reaction until the drying and calcination steps (Figure 2(b6,b7)). This observation is in good agreement with the results of ^29^Si NMR. However, the peak at −9.4 ppm for the -Si-CH_3_ group shifts to −4.0 ppm during the same hydrolysis processes (Figure 2(b2–b5))) due to the substitution of -Si-OCH_3_ by -Si-OH at the neighboring sites, since the -Si-OH shows minor shielding influence. After curing in the gel state, all -Si-OCH_3_ and -Si-OH structures change into the -Si-O-Si- network via condensation, the peak at 49.6 ppm almost disappears, and only the ^13^C peak of -Si-CH_3_ remains intact at −3.5 ppm (Figure 2(b6)). After calcination, the solvent signal almost vanishes, and the only remained signal of -Si-CH_3_ at −3.5 ppm becomes much broader (Figure 2(b7)) due to the increasing rigidity upon the highly cross-linked reaction.

In summary, both ^29^Si and ^13^C NMR spectra reveal that different types of intermediate species (oligomer) are generated during the sol-gel process, thus helping to understand how MTMS evolves into highly cross-linked methyl-modified silica species. The silica microspheres are embedded within the products derived from the hydrolysis and condensation of MTMS, thus leading to MSL formation. The methyl-terminated surface is responsible for the hydrophobic property of the MSL.

### 3.2. Characterization of MSL-Supported α-Al_2_O_3_ Ceramic Membranes

**Figure 3:**
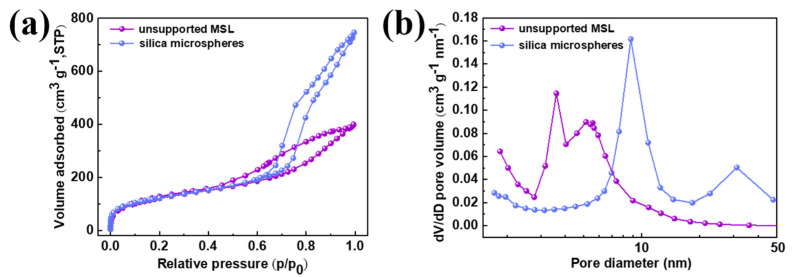
N_2_ adsorption-desorption isotherms (**a**) and pore size distribution (**b**) of the silica microspheres and unsupported MSL.

The MSL was prepared by dispersing silica microspheres with a diameter of approximately 900 nm into the sol derived from MTMS, followed by dip-coating the composite sol on porous α-Al_2_O_3_ ceramic membranes. As shown in Figure 3a, the silica microspheres exhibit a typical type IV N_2_ adsorption isotherm, indicating the presence of mesoporous structure in the sample [46]. A predominant pore size distribution centered at 8.8 nm, accompanied by a secondary peak at 31 nm, is observed in the silica microspheres (Figure 3b). Compared to silica microspheres, the unsupported MSL retains a type IV isotherm, suggesting that the mesoporous structure is preserved within the layer after doping silica microspheres (Figure 3a). As shown in Figure 3b, pores smaller than 3 nm exist within the unsupported MSL, which may originate from the hydrolysis and condensation of MTMS. Additionally, a bimodal pore size distribution is observed at 3.6 and 5.2 nm. It is noticed that the bimodal peaks shift significantly toward smaller diameters in comparison with those of the silica microspheres. This observation deduces that after dispersing silica microspheres into the sol, the silica species (oligomer) in the sol may penetrate the pore channels or accumulate at the pore entrances of the silica microspheres, thus leading to a reduction in pore size, surface area, and pore volume (Table 1). However, the doping of silica microspheres tends to create porosity in the unsupported MSL because the silica microspheres are porous, which may facilitate the separation since the pores provide a pathway for mass transfer.

**Figure 4:**
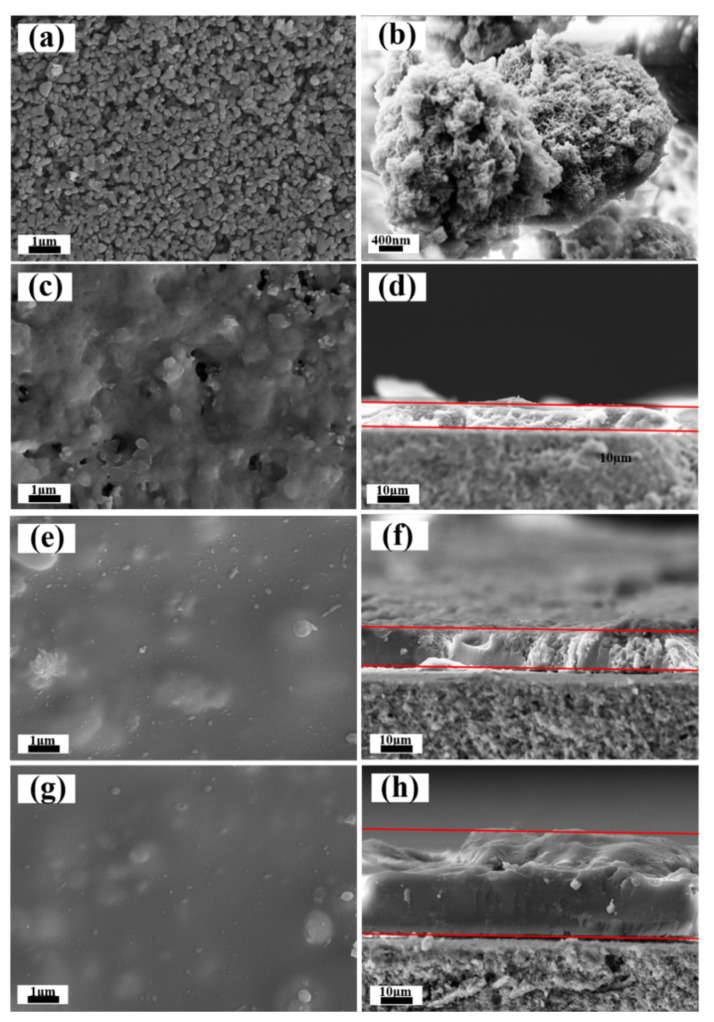
Scanning electron microscopy (SEM) surface morphology of α-Al_2_O_3_ ceramic membranes (**a**), grinded unsupported MSL (**b**), surface morphology of MSL-5 (**c**), MSL-10 (**e**) and MSL-15 (**g**). SEM cross-sectional morphology of MSL-5 (**d**), MSL-10 (**f**) and MSL-15 (**h**).

The surface and cross-sectional morphology of the samples are shown in the SEM images (Figure 4). The pristine α-Al_2_O_3_ ceramic membrane comprises sintered α-Al_2_O_3_ particles, which accumulate and aggregate to form interconnected porosity within the ceramic membrane (Figure 4a). The calcined unsupported MSL is ground for SEM observation. It can be seen from Figure 4b that the doped silica microspheres cannot be distinctly identified because they have been integrated into xerogels. After dip-coating five times (MSL-5), a layer is supported on the α-Al_2_O_3_ ceramic membrane. Still, it does not cover the ceramic completely, as demonstrated by a fraction of the ceramic surface still visible in the SEM image (Figure 4c). Further increasing the dip-coating cycle to 10 (MSL-10) and 15 (MSL-15) results in a continuous and integrated layer, where no obvious defects such as pinholes or microcracks are observed (Figure 4e,g). Obviously, the doped silica microspheres are encapsulated within the layer and seem to bulge from the layer, forming a relatively rough surface. The bulges are less pronounced for the MSL-15 sample (Figure 4g). As seen from Figure 4d,f,h, the thickness of the MSL ranges from 8 to 32 μm, increasing with the increase of dip-coating cycles as expected.

Figure S4 shows the nitrogen fluxes through the pristine α-Al_2_O_3_ ceramic membranes and the MSL-supported samples under a transmembrane pressure of 100 kPa. The pristine α-Al_2_O_3_ ceramic membrane exhibits the maximum nitrogen flux (0.096 × 10^5^ L m^−2^ h^−1^), and the MSL-supported membranes show a decreasing nitrogen flux with increasing dip-coating cycles [47]. The MSL is expected to yield gas resistance in the membranes due to its relatively smaller pore size than that of the pristine α-Al_2_O_3_ ceramic membrane (Figure 3 and Figure S1). Increasing the dip-coating cycles leads to an increase in MSL thickness (Figure 4), thus increasing gas resistance and decreasing the nitrogen flux. The detection of nitrogen flux through the MSL-supported membranes indicates that through-pores rather than dead-end pores are present within the membranes. Through-pores are favorable because they provide a pathway for the transport of VOCs molecules.

**Figure 5:**
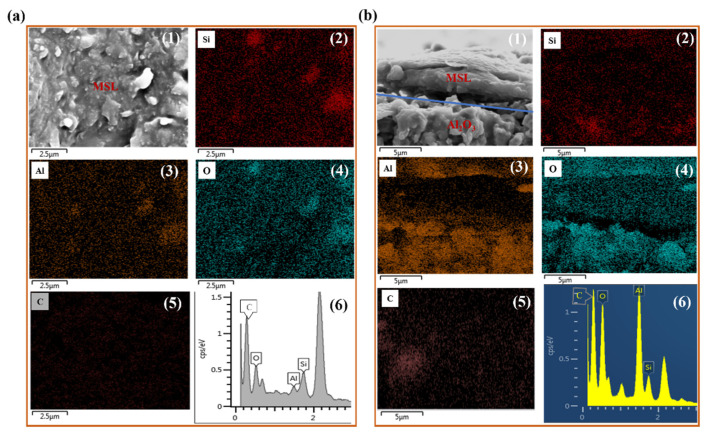
SEM surface (**a**1) and cross-section images (**b**1) of MSL-10; energy-dispersive X-ray spectroscopy (EDS) surface (**a**) and cross-section (**b**) imaging of Si (2), Al (3), O (4), and C (5) element and spectra (6) of MSL-10.

As shown in Figure 5a, both Si and C elements can be detected on the surface of MSL-10 (Figure 5(a2,a5,a6)) via energy-dispersive X-ray spectroscopy (EDS), indicating the uniform distribution of methyl groups on the surface. The percentage of carbon atoms reaches 65.4%, showing a high loading of methyl groups on the MSL-10 surface. The EDS images also confirm the intrusion of the composite sol into the α-Al_2_O_3_ ceramic membrane, which can be verified by the presence of Si and C elements in the supports (Figure 5(b2,b5)). This observation also contributes to the decrease of nitrogen flux in the MSL-10 compared to the pristine α-Al_2_O_3_ ceramic membrane.

Figure S5 shows the wettability and roughness of the pristine α-Al_2_O_3_ ceramic membranes and the MSL-supported samples [48]. The water and MTBE droplets spread rapidly on the surface of the pristine α-Al_2_O_3_ ceramic membrane and are completely adsorbed, reflecting that the α-Al_2_O_3_ ceramic membrane is inherently amphiphilic, with a contact angle of 0° for both water and MTBE (Figure S5a,b). The deposition of MSL on α-Al_2_O_3_ ceramic membranes make the samples change from amphiphilic to hydrophobic and oleophilic, as verified by a water contact angle ranging from 143° to 162° (Figure S5d,g,j) and an MTBE contact angle of 0° (Figure S5e,h,k). The wettability depends on the surface chemical property and surface roughness [49]. The superhydrophilicity of the pristine α-Al_2_O_3_ ceramic membrane generates from surface hydroxyl groups. The hydrophobic property of the MSL-supported samples originates from the methyl groups on the surface of the layers, which have considerably low surface free energy. However, such a surface free energy is not low enough to render oleophobic nature, and accordingly, the sample exhibits an MTBE contact angle of 0°. The AFM images show that the pristine α-Al_2_O_3_ ceramic membrane possesses a root mean square (RMS) roughness of 204 nm (Figure S5c). The RMS roughness decreases to 147 (Figure S5f), 82.3 (Figure S5i), and 72.6 nm (Figure S5l) for the samples MSL-5, MSL-10, and MSL-15, respectively [50]. The α-Al_2_O_3_ ceramic membrane has a rather rough surface since it is fabricated by large α-Al_2_O_3_ powders via dry-pressing and solid-state sintering. The sol, primarily consisting of fine particles, tends to form a smoother surface after gelation, and therefore multiple dip-coating helps to reduce the roughness of the α-Al_2_O_3_ ceramic membrane gradually. It is reasonable that the sample MSL-15 has a smaller WCA (143°) than that of MSL-10 (162°) since the former sample possesses relatively lower RMS roughness. Nevertheless, compared to the sample MSL-10, the sample MSL-5 has a lower WCA despite its higher RMS roughness. This observation may be because MSL-5 fails to cover the hydrophilic α-Al_2_O_3_ ceramic surface totally (Figure 4c,d).

### 3.3. Separation of MTBE from Aqueous Solution

Liquid entry pressure (LEP) is crucial for membrane wetting [51]. The liquid wets the membranes and then passes through the pores of membranes if a transmembrane pressure more enormous than LEP is applied. Figure S6 exhibits a water flux of 5.99 kg m^−2^ h^−1^ in the pristine α-Al_2_O_3_ ceramic membrane at a transmembrane pressure of 89 kPa, suggesting the LEP for water (LEP_w_) is estimated to be 89 kPa. However, the estimated LEP_w_ for the MSL-5, MSL-10, and MSL-15 membrane is 465, 522 and 578 kPa, respectively, considerably higher than the pristine α-Al_2_O_3_ ceramic membrane. This observation is ascribed to the hydrophobic nature and the significantly smaller pore size of the MSL. Since the separation is performed at a transmembrane pressure of approximately 100 kPa, the MSL-supported membranes cannot be wetted by liquid water, which is extremely important for the MTBE/water separation.

**Figure 6:**
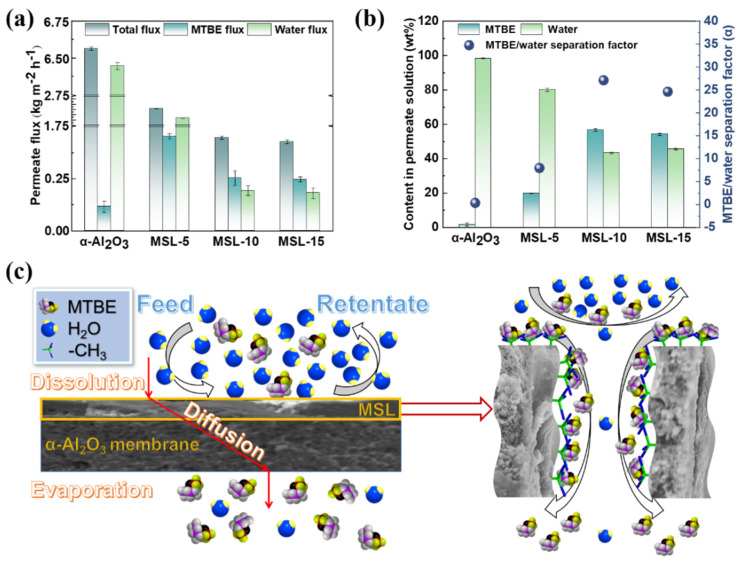
MTBE/water separation performance for the pristine and MSL deposited α-Al_2_O_3_ membranes. Permeate flux (**a**); content in the permeate and separation factor (**b**). Proposed mechanism for the MTBE/water separation by MSL-deposited α-Al_2_O_3_ ceramic membrane (**c**).

As shown in Figure 6a,b, while used in the separation of 4.6 wt% MTBE aqueous solution, the pristine α-Al_2_O_3_ ceramic membrane has a total flux of 6.5 kg m^−2^ h^−1^ and shows an MTBE/water separation factor of only 0.3, clearly revealing that the pristine α-Al_2_O_3_ ceramic membrane fails to separate MTBE from aqueous solution. For the MSL-5 membrane, the total flux through the sample and the water concentration in the permeate flux decrease sharply to 2.3 kg m^−2^ h^−1^ and 80.2%, respectively, but the MTBE/water separation factor increases to 7.9. This observation indicates that the formation of the MSL on α-Al_2_O_3_ ceramic facilitates the MTBE/water separation. Increasing the dip-coating cycle to 10 leads to a decrease in the total flux. Nevertheless, the MTBE flux and the MTBE concentration in the permeate solution exceed those of water, respectively. The MTBE/water separation factor increases dramatically from 7.9 to 27.1, suggesting that more dip-coating cycles are in favor of the MTBE/water separation. However, further increasing the dip-coating cycle to 15 does not further enhance the separation performance, which is evidenced by the fact that the total flux and the water or MTBE flux remain almost constant, and the MTBE/water separation factor decreases slightly to 24.6.

The pristine α-Al_2_O_3_ ceramic membrane manifests an inherently amphiphilic nature and possesses an overwhelmingly larger pore than the molecule size of water and MTBE. Hence, while driven by the vacuum at the permeate side, both liquid water and MTBE can pass through the pristine α-Al_2_O_3_ ceramic membrane, thus giving rise to a relatively poor MTBE/water separation performance. In contrast, the MSL-supported α-Al_2_O_3_ ceramic membranes are selective toward MTBE, and the separation may be realized by pervaporation which complies with the solution-diffusion mechanism. It is generally believed that both fluorinated and nonfluorinated alkyl groups have an affinity toward VOCs. Consequently, MTBE molecules in the aqueous solution are attracted by the methyl groups present in the MSL; in other words, they dissolve easily in the MSL-supported membranes because the methyl groups are amiable to MTBE molecules (Figure 6c). The mesopores within the MSL provide a pathway for the MTBE molecules, which diffuse through the membranes and then vaporize at the permeate side (Figure 6c). The membrane rejects the majority of liquid water due to its hydrophobic nature. The presence of water in the permeate flux may be ascribed to the water vapor, which is generated at the feed solution/membrane interface and then penetrates through the membrane and finally condenses into the permeate flux. A trace of liquid water might also surmount the barrier that originates from the hydrophobic layer and diffuses across the membrane [52], which is another reason for detecting water in the permeate flux. In our previous work, mesoporous silica membrane modified by alkyl groups was used for MTBE/water separation. This work obtains a higher total flux and a considerably larger MTBE/water separation factor compared to our previous work. This observation may be attributed to the possibility that the MSL possesses a higher loading of methyl groups, either on the outside surface or on the inner pore surface, compared to the membrane modified by a simple post-grafting approach in the previous work [35]. In the post-grafting method, a condensation reaction occurs between the surface hydroxyl groups on the membranes and the alkoxy groups in the modifier. The loading of the functional group (i.e., a methyl group) depends on the number of surface hydroxyl groups. At least three surface hydroxyl groups are required to firmly anchor one modifier molecule via covalent bonds to the membrane if adopting trialkoxysilane (i.e., methyltrimethoxysilane, MTMS) as a modifier [53]. The maximum loading of the methyl group per mole silica membrane that can be achieved by the post-grafting approach is 1/3 mole, even if one hydroxy group is present in every silicon atom in the silica membrane [54]. Actually, the loading of the methyl group in a mole silica membrane is considerably less than 1/3 mole because most silicon atoms in silica membranes do not link to hydroxy groups [55]. For the one-pot synthesis approach, however, one mole unsupported MSL, in which the doping silica microspheres are negligible due to the low concentration, contains one mole methyl group since MTMS is used for both the precursor and modifier for the membranes. Therefore, the one-pot synthesis approach leads to higher loading of alkyl groups than the post-grafting method. As discussed in Figure 4, the hydrophobic MSL does not entirely cover the hydrophilic α-Al_2_O_3_ ceramic surface in the MSL-5 membranes. Accordingly, liquid water may pass through these membranes via the residual hydrophilic surface, resulting in a relatively lower MTBE/water separation factor for the MSL-5 sample than the MSL-10 and MSL-15 membranes (Figure 6b).

**Figure 7:**
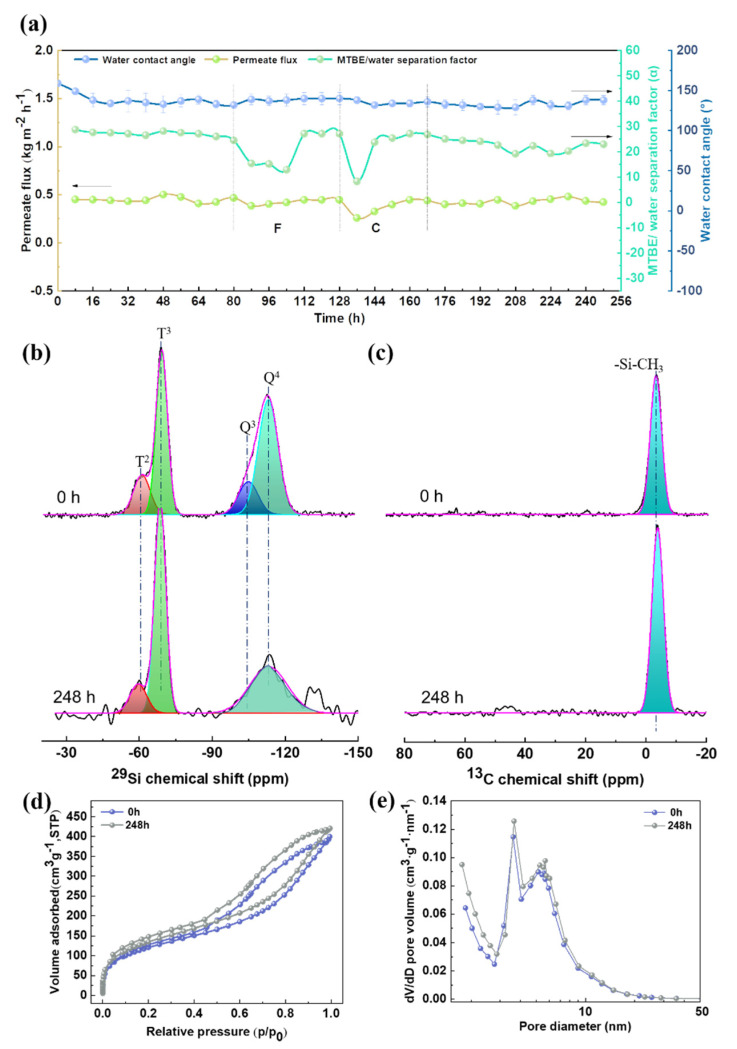
Long-term stability of the MSL-10 membrane operating under the operating conditions as follows: a feed temperature of 30 °C, an MTBE concentration of 4.6 wt%, and a feed flow rate of 200 mL min^−1^, except for the F and C regions, where region F indicates that the measurement is conducted by varying the feed flow rate and region C represents the variation of the MTBE content in feed solution (**a**). Solid state ^29^Si MAS (**b**) and ^13^C MAS (**c**) NMR spectra, N_2_ adsorption-desorption isotherms (**d**), and pore size distribution (**e**) of the unsupported MSL after exposure to the MTBE aqueous solution for different times.

Figure 7 shows the long-term stability of the MSL-10 membrane during the separation of MTBE from an aqueous solution. The investigation is conducted under a flow rate of 200 mL min^−^^1^, a feed concentration of 4.6 wt%, and a feed temperature of 30 °C, unless otherwise stated. In the first 16 h, the water contact angle decreases from 159° to 138° and stays almost unchanged. A few encapsulated silica microspheres, especially those embedded just beneath the superficial surface of MSL, may escape from the layer due to the scouring effect imposed by the feed solution in the initial stage. This observation leads to a decrease in membrane surface roughness and consequently a reduction in WCA. The total flux and the MTBE/water separation factor are maintained at approximately 0.448 kg m^−2^ h^−1^ and 27 for 80 h, respectively. In order to reveal the influence of operating conditions on the long-term stability of the membranes, the experiment is conducted by changing the MTBE concentration and the feed flow rate, and the investigation lasts for another 80 h (see Figure S7 in the Supplementary Material for a detailed discussion). The results show that the total flux and MTBE/water separation factor are restored to the original level when adjusting the operating conditions to the initial stage. Afterward, the separation continues for another 104 h, and it is found that the separation performance remains almost constant until the experiment finishes at 248 h. This observation reveals that the membranes are highly stable under the experimental conditions.

As discussed above, the separation performance may depend on the surface methyl groups and the pore structure of MSL [56]. The former renders affinity to attract MTBE molecules from the aqueous solution, and the latter provides a pathway for the diffusion of MTBE molecules across membranes. To examine the MSL layer’s chemical structure and pore structure after long-term separation, the unsupported MSL is exposed to the MTBE aqueous solution (4.6 wt% MTBE, 30 °C) for a different time, and then submitted to NMR measurement and N_2_ adsorption after drying. The ^29^Si MAS NMR spectra of the pristine unsupported MSL and the MSL after exposure to MTBE aqueous solution for 248 h, fitted by the Gaussian approach, are shown in Figure 7b. Integrating the Q^m^ and T^n^ resonances can quantitatively calculate the relative amount (mol%) of *T*^2^, *T*^3^, *Q*^3^[Si(OSi)_3_OH], and *Q*^4^[Si(OSi)_4_] silicon atoms, and the results are listed in Table 2. The absolute values (mmol g^−1^) for the methyl groups on the surface of MSL can be calculated according to the following equation [40]. One mole unsupported MSL is used for the calculation.

(3) $$\left[CH_{3}\right]=\frac{I_{T^{2}}+I_{T^{3}}}{60\times I_{Q^{4}}+69\times I_{Q^{3}}+67\times I_{T^{3}}+76\times I_{T^{2}}}\times 1000\;$$

where the variable *I* means the molar concentration (mol%) of *Q*^m^ (n = 3–4) and *T*^n^ (m = 2–3) species, the denominator represents the molecular mass (g mol^−1^) of silica membranes, and the numerator denotes the molar percentage of methyl groups.

Table 2 and Figure 7b clearly show that the intensity of *Q* resonance decreases, and the concentration of surface methyl groups increases from 7.1 to 9.5 mmol g^−1^ after exposure for 248 h. As mentioned above, the *Q*^m^ signals originate from the doping silica microspheres. The decrease in *Q*^m^ intensity reveals that a fraction of doping silica microspheres may break away from the MSL after exposure. This observation agrees with the discussion mentioned above on the decrease of WCA in the long-term separation experiment. As exhibited in the ^13^C MAS NMR spectra (Figure 7c), the intensity of methyl groups obviously increases after exposure, consistent with the ^29^Si MAS NMR measurement. The partial escape of silica microspheres from MSL contributes to the enhancement of the signals related to methyl groups. Both ^29^Si and ^13^C NMR spectra confirm that the methyl groups remain intact after exposure for 248 h. As shown in Figure 7d,e, the pore structure of the unsupported MSL can also be maintained after exposure for 248 h. Figure S8 shows the surface morphology and nitrogen flux of the pristine MSL-10 membrane and the one after separation for 248 h. The morphology remains almost unchanged, except that the aggregation of silica microspheres seems to be less pronounced after long-term separation (Figure S8a,b). Although the nitrogen flux decreases from 0.047 × 10^5^ L m^−2^ h^−1^ to 0.034 × 10^5^ L m^−2^ h^−1^ after 248 h operation (Figure S8c), the permeate flux remains stable at 0.448 kg m^−2^ h^−1^ (Figure 7a). The intact surface methyl groups and the stable pore structure are responsible for the outstanding long-term stability of the membranes.

Table 3 compares the MTBE/water separation performance of various membranes. It should be mentioned that the separation performance of our membranes is inferior to that of some polymer membranes (e.g., PERVAP-1060 and M10/MFFK) and ceramic membranes (e.g., TiO_2_-5KD and ZrO_2_-5KD). However, our membranes are competitive with mesoporous Al_2_O_3_ and ZrO_2_ ceramic membranes. It is noteworthy that the separation performance in this work is remarkably superior to that of our previous work, where alkyl groups are adopted to modify the silica membranes via a post-grafting method. More importantly, the utilization of nonfluorinated alkyl groups instead of fluorocarbon groups is of great significance since the absence of fluoride is highly advantageous to environmental protection.

## 4. Conclusions

The methyl-modified silica layer (MSL) has been successfully supported on porous α-Al_2_O_3_ ceramic membranes by the sol-gel and dip-coating method using environmentally friendly nonfluorinated alkylsilane, methyltrimethoxysilane (MTMS), as a precursor. The doping of pre-synthesized mesoporous silica microspheres leads to a bimodal pore size distribution, centered at 3.6 and 5.2 nm, respectively, for the unsupported MSL, and a rough surface for the supported MSL. The amphiphilic α-Al_2_O_3_ ceramic membranes are converted into hydrophobic and oleophilic after the deposition of MSL, showing a water contact angle ranging from 143° to 162° and an MTBE (methyl tert-butyl ether) contact angle of 0°. When used to separate MTBE from aqueous solution, the MSL-supported α-Al_2_O_3_ ceramic membranes (MSL-10) exhibit an MTBE/water separation factor of 27.1 and a total flux of 0.448 kg m^−2^ h^−1^. The MTBE/water separation performance has been considerably enhanced compared to other NFAS-modified membranes ever reported. The membranes also show outstanding long-term stability, with a stable MTBE/water separation factor and total flux even after operation for more than 248 h.

## Figures and Tables

**Table 1:** Pore structure parameter of silica microspheres and unsupported MSL.

Samples	Surface Area (m^2^ g^−^^1^)	Pore Volume (cm^3^ g^−1^)	Pore Size (nm)
Silica microspheres	736.0	1.3	8.8 and 31
Unsupported MSL	447.2	0.6	3.6 and 5.2

**Table 2:** Deconvolution results of solid state ^29^Si MAS NMR spectra of the unsupported MSL exposure to MTBE aqueous solution for different times.

	*Q*^4^ (mol%)	*Q*^3^ (mol%)	*T*^3^ (mol%)	*T*^2^ (mol%)	[*CH*_3_] (mmol g^−1^)
0 h	42.5	11.0	34.2	12.3	7.1
248 h	37.8	0.0	51.1	11.1	9.5

**Table 3:** MTBE/water separation performance of various membranes.

Membranes	CM (wt%)	T (°C)	J (kg m^−2^ h^−1^)	α	Modifier	Refs.
PEBAX-4033	1.0	40	0.03	33.0	-F	[57]
PERVAP-1060	1.0	40	0.70	270.0	-F	[57]
PERVAP-1070	1.0	40	0.25	280.0	-F	[57]
M10/MFFK	1.0	50	0.82	310.0	-CH_3_	[16]
Al_2_O_3_-5nm	1.0	35	0.70	1.1	FAS	[20]
TiO_2_-5KD	1.0	35	1.95	84.0	FAS	[20]
ZrO_2_-5KD	1.0	35	1.65	56.0	FAS	[20]
ZrO_2_-3nm	1.5	35	0.50	15.0	FAS	[19]
ZrO_2_-200nm	1.5	35	7.00	27.0	FAS	[19]
(0.005PFOTES)SiO_2_	3.0	40	0.35	24.6	FAS	[35]
(0.005TFPTES)SiO_2_	3.0	40	0.31	19.1	FAS	[35]
(0.005OTES)SiO_2_	3.0	40	0.32	15.3	OTES	[35]
(0.005PTES)SiO_2_	3.0	40	0.39	12.0	PTES	[35]
MSL-10	4.6	30	0.45	27.1	MTMS	This work

Note: CM represents the content of MTBE in the feed solution. T refers to the feed temperature. J and α denote the total flux and MTBE/water separation factor, respectively.

## Data Availability

Not applicable.

## Supplementary Materials

The following supporting information can be downloaded at: https://www.mdpi.com/article/10.3390/membranes12050452/s1. Description of the preparation of porous α-Al_2_O_3_ ceramic membranes and pore size distribution (Figure S1); Experimental setup for pervaporation (Figure S2); Structural and performance characterization of MSL-supported membranes (Figure S3–S8).
