# Supplementary material for: A Methyl-Modified Silica Layer Supported on Porous Ceramic Membranes for the Enhanced Separation of Methyl Tert-Butyl Ether from Aqueous Solution
Source: Membranes (Basel). 2022 Apr 22;12(5):452. doi: 10.3390/membranes12050452 (PMC9144733; doi:10.3390/membranes12050452)
Supplement: Supplementary file 1 [file membranes-12-00452-s001.zip › membranes-1672542-supplementary.pdf]

A Methyl-Modified Silica Layer Supported on Porous Ceramic Membranes for the Enhanced Separation of Methyl Tert-Butyl Ether from Aqueous Solution

Ligang Xu [1], Yali Wang [1,*], Qunyan Li [1], Suping Cui [1], Mingxue Tang [2], Zuoren Nie [1] and Qi Wei [1,*]

[1] Faculty of Materials and Manufacturing, Beijing University of Technology, 100 Pingleyuan, Chaoyang District, Beijing 100124, China; xulg@emails.bjut.edu.cn (L.X.); qyli@bjut.edu.cn (Q.L.); cuisuping@bjut.edu.cn (S.C.); zrnie@bjut.edu.cn (Z.N.)

[2] Center for High Pressure Science and Technology Advanced Research, Beijing 100094, China; mingxue.tang@hpstar.ac.cn

* Correspondence: wangyali1978@bjut.edu.cn (Y.W.); qiwei@bjut.edu.cn (Q.W.); Tel.: +86-10-6739-6085 (Y.W.); +86-10-6739-6206 (Q.W.)

Supplementary Material S1. Preparation of porous α-Al_2O_3 ceramic membranes

Porous α-Al_2O_3 ceramic membranes were prepared using a dry-pressing and solid-state sintering method with water-soluble starch as a pore-forming agent. Aluminum oxide was mixed with starch in a mass ratio of 3:1. Then, 40 g of the mixture was dispersed into 20 ml deionized water and wet-milled for 10 h by a ball mill to form a slurry. The slurry was dried at 60 °C for more than 12 h to remove the water. After crushing and sieving to remove coarse particles larger than 200 mesh, the dried powder was uniaxially pressed under 20 MPa in a cylindrical mold (φ=25 mm) to obtain planar green bodies. The green bodies were sintered at 500 °C for 4 h to remove starch and then at 1290 °C for another 12 h with a ramping rate of 1 °C min^{-1}. The sintered ceramic membranes were ultrasonically cleaned twice, each for 5 min, and then dried at 80 °C for 12 h until further use.

The pore size distribution of the cordierite membranes is shown in `Figure S1`. The pristine α-Al2O3 ceramic membrane exhibits a predominant pore size distribution centered at approximately 36 nm.

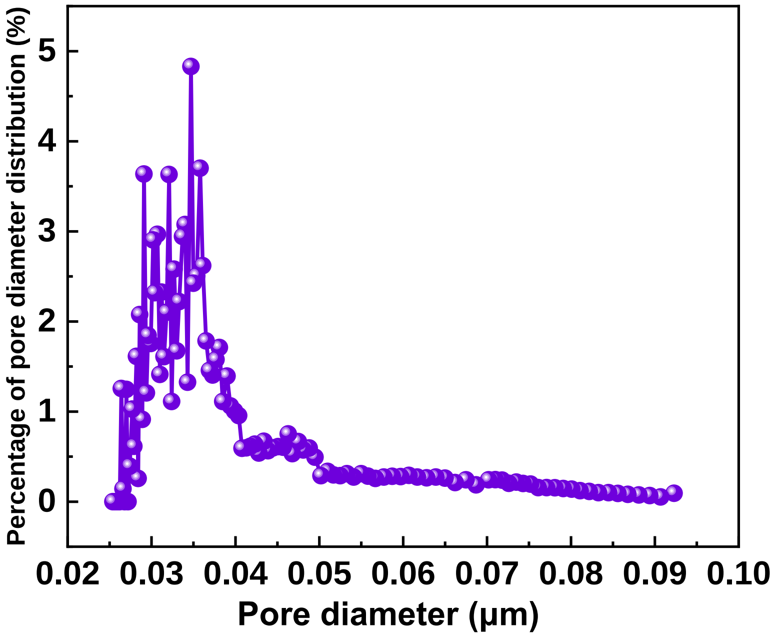

Figure S1. Pore size distribution of α−Al_2O_3 ceramic membrane.

Supplementary Material S2. Experimental setup for pervaporation

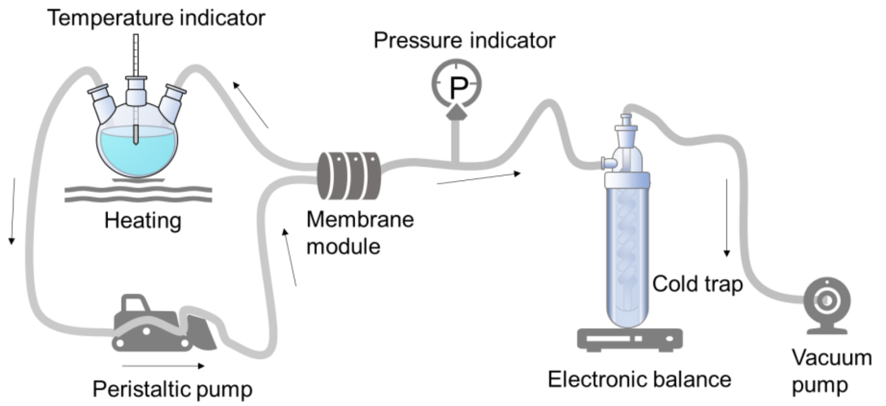

Figure S2. Schematic representation of the setup for pervaporation.

Supplementary Material S3. Evolution of the Spatial Structure of the Products at Different Preparation Steps

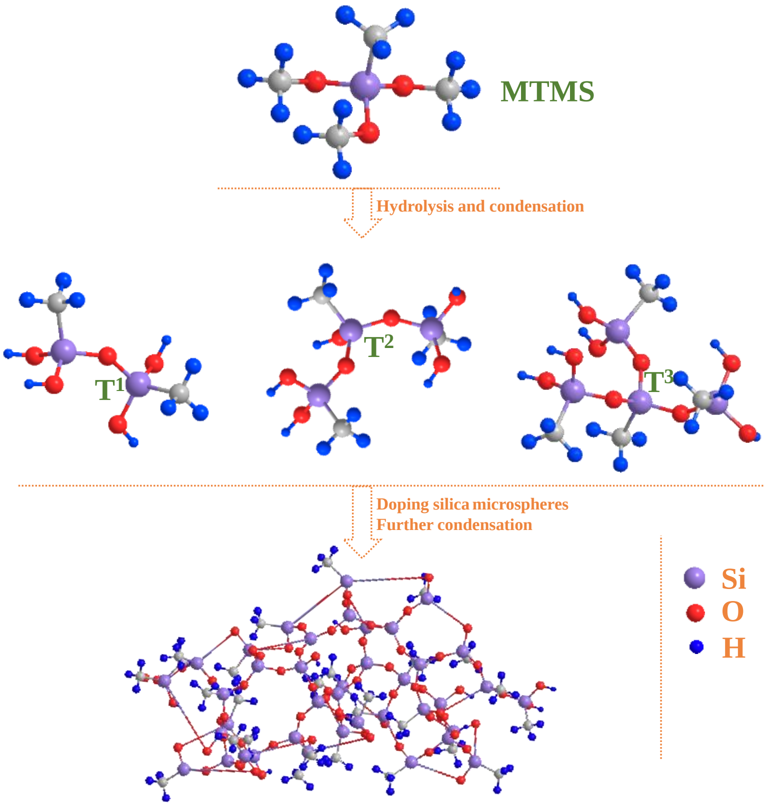

Figure S3. Illustration of the spatial structure during the sol−gel reaction procedure. MTMS (methyltrimethoxysilane) precursor (**a**); oligomer (**b**); highly cross−linked methyl-modified silica species (**c**).

Supplementary Material S4. Nitrogen fluxes in the pristine α-Al2O3 ceramic membranes and the MSL-supported samples

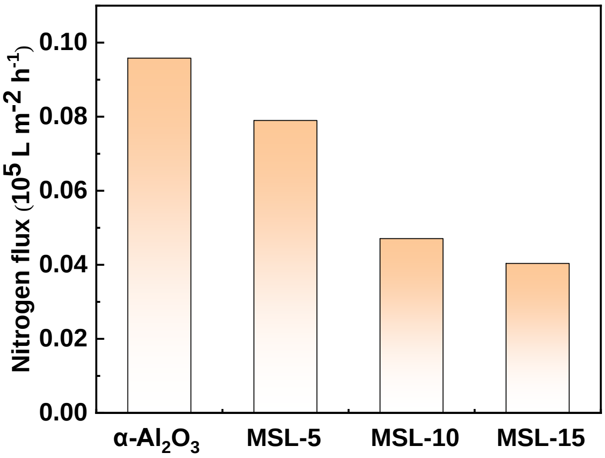

Figure S4. Nitrogen fluxes in the pristine α−Al_2O_3 ceramic membrane and the MSL (methyl-modified silica layer)−supported samples under a transmembrane pressure of 100 kPa.

Supplementary Material S5. Wettability and roughness of the pristine α-Al2O3 ceramic membranes and the MSL-supported samples

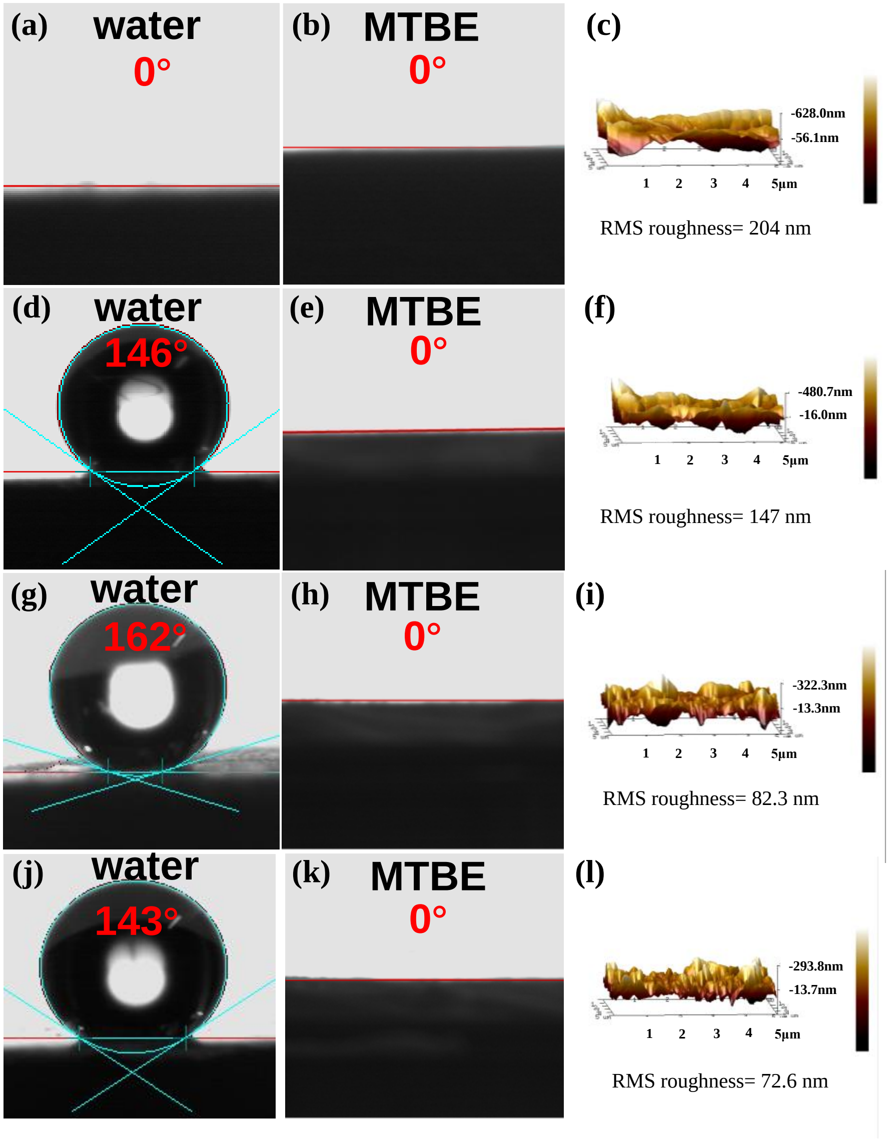

Figure S5. Water (**a, d, g, j**) and MTBE (methyl tert−butyl ether) (**b, e, h, k**) contact angles and AFM (atomic force microscopy) images (**c, f, i, l**) of the pristine α−Al_2O_3 ceramic membrane (**a, b, c**), MSL (methyl-modified silica layer)−5 (**d, e, f**), MSL−10 (**g, h, i**) and MSL−15 (**j, k, l**) membranes.

Supplementary Material S6. Water flux and LEP

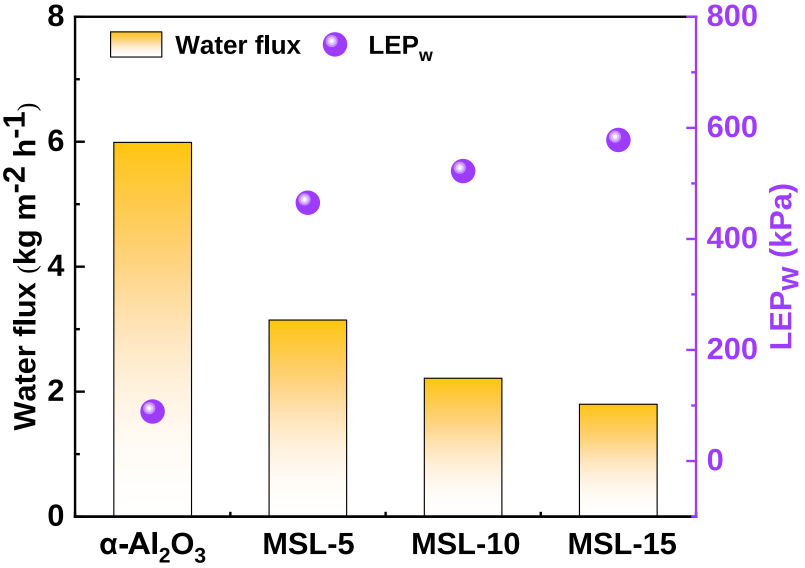

Figure S6. Water fluxes at the LEP (liquid entry pressure) of the pristine and MSL (methyl-modified silica layer)–supported α–Al_2O_3 ceramic membranes.

Supplementary Material S7. Influence of operating conditions on the MTBE/water separation performance

For the MSL-10 membrane, the influence of MTBE content in the feed solution on the separation performance is shown in Figure S7a,b. With the increase of MTBE content, the total flux and MTBE flux increase monotonically because the increasing MTBE concentration promotes the driving force for the MTBE transport through the membrane. In comparison with MTBE, the water flux decreases extremely slightly because of the negligible change of the water concentration from 99 to 95.4wt%. As shown in Figure S7b, the MTBE/water separation factor increases gradually from 8.3 to 27.1 when the MTBE content in the feed solution increases from 1 to 4.6wt%. It is noticed that MTBE has been intensely concentrated to as high as 56.7% in the permeate solution compared to feed solution (4.6wt% MTBE) after the separation process, demonstrating an efficient separation. The influence of the feed flow rate on the separation performance of the MSL-10 membrane is shown in Figure S7c,d. It can be seen that the total flux increases gradually with increasing feed flow rate, and the MTBE/water separation factor rises sharply to 27.1 at the feed flow rate of 200 mL min-1. It is possible that at a specific high feed flow rate, the MTBE molecules have more opportunities to reach the membrane surface because of the higher motion velocity, and they then dissolve in the membrane, thus resulting in an enhanced separation factor. As shown in Figure S7d, the concentration of MTBE exceeds that of water in the permeate flux at the feed flow rate of 200 ml min-1, implying an effective MTBE/water separation. The influence of the feed temperature on the separation performance of the MSL-10 membrane is shown in Figure S7e,f. It is found that both MTBE flux and MTBE/H2O separation factor decrease slightly with increasing temperatures. This observation may be attributed to the fact that higher temperatures help to promote the vaporization of water, leading to an increase in the water flux and therefore a decrease in the MTBE/H2O separation factor.

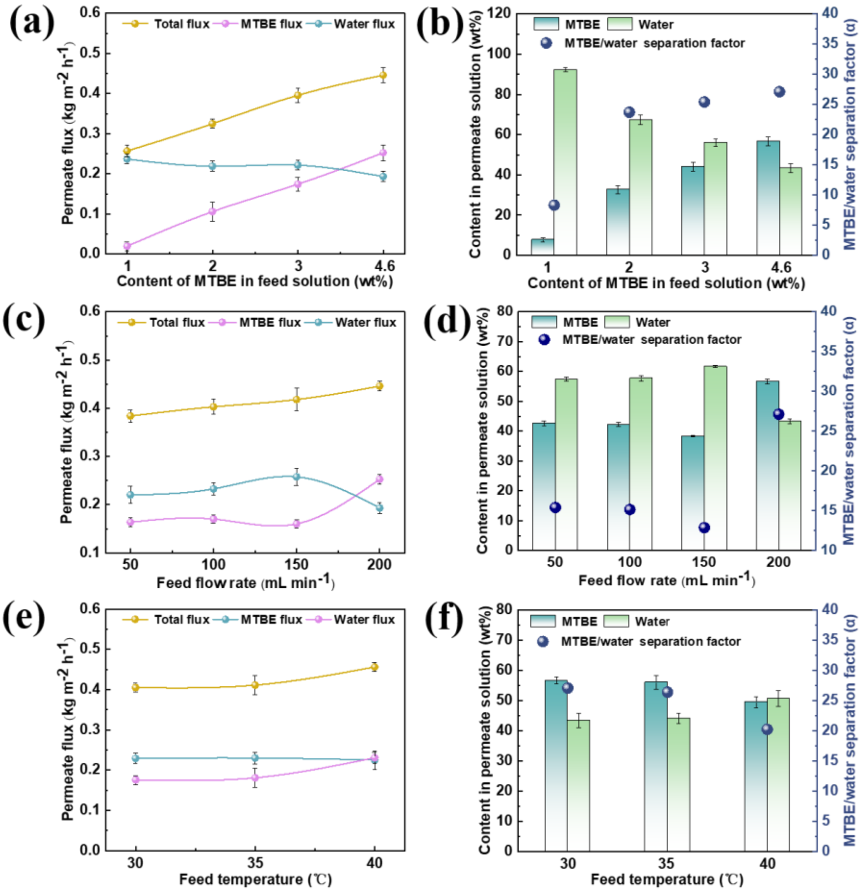

Figure S7. Effect of the MTBE (methyl tert–butyl ether) content in the feed solution (**a, b**), the feed flow rate (**c, d**) and the feed temperature (**e, f**) on the permeate flux (**a, c, e**) and content in permeate solution and MTBE/separation factor (**b, d, f**) of the MSL (methyl-modified silica layer)–10 membrane.

Supplementary Material S8. Change in morphology and N2 permeance after long-term separation

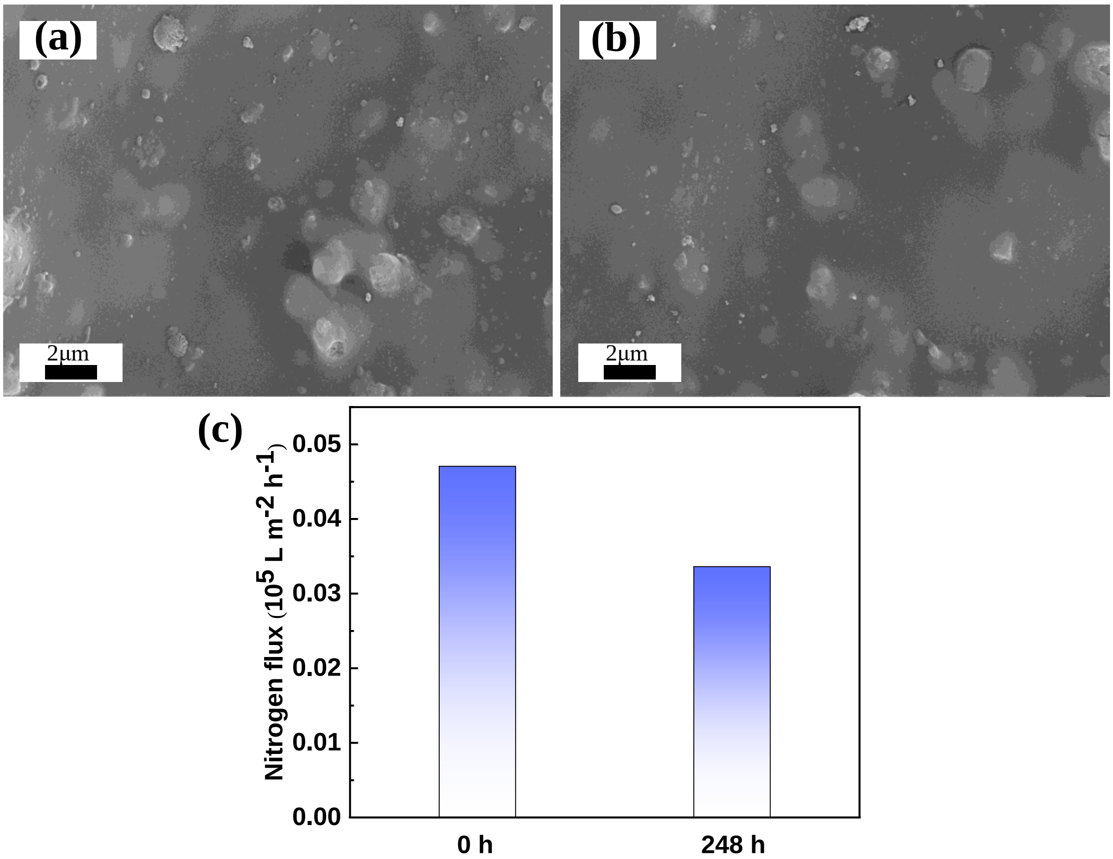

Figure S8. SEM (Scanning electron microscopy) images and nitrogen fluxes of the MSL (methyl-modified silica layer)−10 membrane after operation for different times,
